# Prognostic Significance of Erythrocyte Sedimentation Rate for Survival in Equine Colic

**DOI:** 10.3390/ani16030476

**Published:** 2026-02-03

**Authors:** Federica Meistro, Riccardo Rinnovati, Edoardo Blanc, Priscilla Berni, Silvia Napoli, Elisa Marcucci, Paola D’Angelo, Marco Ruggeri, Alessandro Spadari, Rodolfo Gialletti

**Affiliations:** 1 Department of Veterinary Medical Sciences, University of Bologna, 40064 Ozzano dell’Emilia, BO, Italy; federica.meistro@unibo.it (F.M.); elisa.marcucci3@unibo.it (E.M.); paola.dangelo7@unibo.it (P.D.); alessandro.spadari@unibo.it (A.S.); 2 Department of Veterinary Science, University of Parma, Via del Taglio 10, 43126 Parma, PR, Italy; edoardo.blanc@unipr.it (E.B.); priscilla.berni@unipr.it (P.B.); silvia.napoli@unipr.it (S.N.); marco.ruggeri@unipr.it (M.R.); rodolfo.gialletti@unipr.it (R.G.)

**Keywords:** equine colic, erythrocyte sedimentation rate, prognosis, survival, biomarker, colic surgery

## Abstract

Accurate early prognostic assessment is a central aspect of clinical decision-making in horses presenting with colic. Despite the availability of multiple clinical and laboratory parameters, reliable biomarkers that provide useful information at the time of admission remain limited. The erythrocyte sedimentation rate is a non-specific indicator of systemic inflammation that has recently been validated for automated point-of-care use in horses. Its behaviour in equine colic has not been previously characterised. This study included 85 horses admitted for colic at two university referral hospitals. The erythrocyte sedimentation rate was measured at admission in all horses and again approximately 24 h after surgery in a subset of surgical cases. Horses were classified as medically managed, surgical survivors or surgical non-survivors. Group comparisons were performed using non-parametric statistical methods, and associations with survival were evaluated. The admission erythrocyte sedimentation rate was significantly lower in surgical non-survivors compared to the other groups (*p* < 0.05). Erythrocyte sedimentation rate rose in surviving horses within 24 h, while it barely changed among non-survivors, and there were no significant changes among surgical values. Actually, these observations suggest that low erythrocyte sedimentation rates on admission are associated with low chances of survival for horses undergoing colic surgery and thus make these rates a feasible additional prognostic indicator for equine colic.

## Simple Summary

Colic is one of the most common and severe emergencies in horses and continues to represent a major cause of death in equine practice. One of the main clinical challenges is identifying, as early as possible, which horses are at greater risk of deterioration or death, particularly at the time of hospital admission when rapid decisions are required. Although several biomarkers are used to support prognostic evaluation, many of them increase slowly and may be of limited value during the early stages of severe disease. In this study, we investigated the erythrocyte sedimentation rate, a non-specific indicator of systemic inflammation widely used in human medicine and recently made available for routine use in horses through automated analysis. This parameter was measured at admission in horses presenting with colic and, in surgical cases, again 24 h later. Horses that did not survive showed markedly lower values at presentation compared to survivors. These results suggest that low erythrocyte sedimentation rate values at admission may be associated with severe systemic compromise. Given that this test is rapid, inexpensive and easy to perform, it may represent a valuable additional tool to support early prognostic assessment in horses with colic.

## 1. Introduction

Colic is one of the major causes of morbidity and mortality in the domestic equine population, and is widely recognised both by owners and veterinarians as a potentially life-threatening emergency because of the sudden onset and rapid clinical evolution [1,2]. Despite advances in diagnostics and perioperative care, colic continues to be a major reason for equine hospital admissions [3]. Several epidemiological studies consistently describe its complex and multifactorial nature, with contributions from management practices, feeding behaviour, environmental influences, and individual susceptibility [4,5]. This heterogeneity is a reason for the considerable variability in reported outcomes, particularly in horses requiring exploratory laparotomy, where survival rates still show substantial variation between centres and among different case types [6,7,8,9].

In this setting, one of the greatest challenges in equine emergency medicine is to determine, at the time of admission, which horses will survive and which are at an increased risk of deterioration or death. Traditional clinical markers, such as heart rate, mucous membrane colour, capillary refill time and pain score, are invaluable for the initial assessment [10]. However, several recent studies have shown that these variables may change substantially over time and may not always be indicative of the extent of underlying gastrointestinal compromise [11,12]. This is particularly evident in horses with strangulating small intestinal lesions or rapidly evolving ischaemia [13,14].

Consequently, increasing attention has been directed toward laboratory biomarkers that can offer timely and objective assessments of disease severity. Blood lactate has become one of the most studied parameters, with several recent analyses confirming its association with lesion type, systemic hypoperfusion, and survival probability in both medical and surgical colic [15,16]. Acute-phase proteins such as serum amyloid A (SAA) and fibrinogen have been investigated for diagnostic and prognostic value and are useful for identifying inflammatory abdominal disease and monitoring its progression [17,18]. However, their relatively slow kinetics limit their utility during the hyperacute stage of severe gastrointestinal compromise, when early decisions are critical [19]. This emphasises the need for additional biomarkers that are rapid, inexpensive, easy to obtain, and that can provide informative prognostic information early during the clinical evaluation.

In this context, the erythrocyte sedimentation rate (ESR) is gaining renewed interest. ESR is a non-specific marker for inflammation, which is widely used in human medicine as a broad indicator of systemic inflammation, and reflects the combined effect of fibrinogen concentration, immunoglobulins, and erythrocyte aggregation dynamics [20,21,22]. Although early veterinary work explored ESR in horses [23], its clinical use declined over time due to the practical limitations of manual Westergren-based techniques that were slow, operator-dependent, and unsuited for emergency settings [24]. A major step forward was achieved with the recent analytical validation of the MINI-PET automated ESR device for equine blood, showing strong agreement with the reference method, high precision, and excellent usability at the point of care [25].

In human patients, ESR typically rises over a 24 h period as acute-phase proteins increase, which has contributed to its role as an indicator of disease progression rather than an early or immediate marker of acute inflammation [26]. In horses, species-specific reference intervals for ESR have now been established in healthy individuals [25], yet no information is currently available on ESR behaviour in equine colic emergencies. In particular, no studies have described how ESR behaves across different forms of colic, whether admission values correspond to disease severity or survival, or whether early changes during the first hours of hospitalisation might carry prognostic significance. Given the clinical importance of early triage, the recognised need for complementary biomarkers in equine colic, and the absence of previous information on the diagnostic or prognostic behaviour of ESR in this context, the present study was designed to investigate whether ESR measured at admission, and its evolution over the first 24 h, may provide clinically relevant insight into survival outcomes in horses presenting with colic syndrome.

## 2. Materials and Methods

### 2.1. Study Design and Objectives

This was a prospective observational study conducted at the Equine Teaching Hospitals of the Universities of Bologna and Parma that involved horses admitted for colic between January 2024 and December 2025. Since the aim of the study was to describe the clinical behaviour of ESR in a true-to-life population of horses presenting with abdominal pain, no inclusion or exclusion criteria were applied other than confirmation of a clinical diagnosis of colic. This ensured the study population mirrored the natural heterogeneity of colic cases that are encountered in referral practice, which range from conditions managed successfully medically to severe gastrointestinal lesions that necessitated urgent surgical intervention.

The primary objective of this study was to determine whether ESR measured at the time of hospital admission is associated with survival in horses presenting with colic, with particular attention to those undergoing exploratory celiotomy. Because colic includes a wide range of gastrointestinal pathologies with different degrees of severity, we also aimed to compare admission ESR values between medically managed horses and those requiring surgical intervention, in order to evaluate whether baseline ESR differs across colic severities that ultimately lead to different survival outcomes. In the subgroup of surgical patients, where a second ESR measurement could be obtained as part of postoperative monitoring, we further investigated whether short-term changes in ESR during the first 24 h of hospitalisation were related to survival. As an exploratory component, we also assessed whether admission ESR differed between horses with small-intestinal and large-intestinal lesions, given the well-recognised differences in prognosis between these two categories of colic. Horses were enrolled prospectively upon presentation to the hospital, and all data were collected as part of routine clinical examination and diagnostic work-up. Blood sampling for ESR required no additional venipuncture. All diagnostic and therapeutic decisions, including whether to pursue medical treatment, perform exploratory celiotomy, or recommend euthanasia on welfare grounds, were made entirely at the discretion of the attending clinicians and were not influenced by the aims of the study. No procedure was performed specifically for research purposes, and patient management reflected real clinical conditions.

### 2.2. Group Classification According to Treatment and Outcome

For the purposes of this study, horses were classified retrospectively into groups according to the clinical pathway they ultimately followed during hospitalisation. Two principal groups were defined. The **medically managed survivor group** consisted of horses admitted for colic that were managed medically and did not undergo exploratory celiotomy. The **surgical group** included horses that required exploratory celiotomy. Within both groups, the intestinal segment affected (small or large intestine) was recorded based on the diagnosis established through physical examination, ultrasonography and, when applicable, surgical findings.

All horses underwent blood sampling, and ESR at admission (ESR(T0)) was obtained. The **medically managed survivor group** contributed only to ESR(T0) values. In contrast, for the surgical group, a second blood sample was collected approximately 24 h after celiotomy, and was used to determine the 24 h ESR (ESR(T24)). Horses that deteriorated or were euthanised before the 24 h postoperative time point did not contribute a postoperative ESR measurement. This **subgroup** included cases for which surgery was initially indicated, but euthanasia was elected before induction of anaesthesia due to poor prognosis, horses euthanised intraoperatively, and horses that underwent celiotomy but died or were euthanised within the first 24 h following surgery. As a consequence, ESR(T24) measurements were available only for horses that survived long enough to undergo routine postoperative blood sampling. Horses euthanised before anaesthesia, euthanised intraoperatively, or that died or were euthanised within the first 24 h after surgery contributed only ESR(T0) values.

Signalment variables (age, sex, breed and intended use) were also recorded. The final outcome for each horse, survival to discharge or non-survival, was documented at the end of hospitalisation and served as the primary endpoint.

### 2.3. ESR Measurement Procedure

ESR determination was performed using the MINI-PET automated analyser (DIESSE Diagnostica Senese S.p.A.; Monteriggioni (SI), Italy), following the analytical procedure validated for equine blood by Pieroni et al. (2023) [25]. Blood samples were collected into 3.0 mL K3-EDTA tubes (Vacutest KIMA, Arzergrande, Padua, Italy), which are routinely used for haematology in both participating hospitals. According to the manufacturer’s instructions, the tubes were gently inverted before analysis to ensure homogeneous erythrocyte resuspension.

The MINI-PET system employs a modified Westergren optical method that allows ESR to be measured directly within the original EDTA tube, without any additional manipulation or transfer of the sample. This photometric technique provides a rapid analytical turnaround, with an optimal reading time of approximately eight minutes for equine blood, enabling ESR to be obtained promptly during emergency evaluation.

In accordance with the stability data reported by Pieroni et al. [25], ESR values in EDTA blood samples remain reliable for 8 h when stored at room temperature, and for 24 h when refrigerated at 4 °C. All analyses in the present study were performed within these established stability intervals to ensure analytical accuracy.

Admission ESR values (ESR(T0)) were obtained for all horses. In cases undergoing celiotomy, when a second EDTA sample was collected approximately 24 h postoperatively as part of routine monitoring, this sample was used to determine the 24 h ESR (ESR(T24)). All ESR measurements were obtained using the same analytical protocol, ensuring consistency across horses and between institutions.

### 2.4. Statistical Analysis

Statistical analyses were conducted to describe ESR behaviour across the study population and to evaluate its potential association with clinical outcome. Continuous variables were assessed for normality using the Kolmogorov–Smirnov test; data not conforming to a normal distribution were summarised as median and interquartile range (IQR), whereas normally distributed variables were expressed as mean ± standard deviation (SD). Categorical data, including sex, breed and lesion location, were reported as counts and proportions.

The analytical approach followed a structured sequence. As an initial step, ESR values at admission (ESR(T0)) were compared between the **medically managed survivor group** and the surgical group in order to explore whether the case management pathway was associated with distinct ESR profiles at presentation. The analysis then focused specifically on the surgical group, where admission ESR values were compared between horses that survived to discharge and those that did not, to determine whether ESR(T0) carried prognostic relevance.

In horses that underwent surgery and for which a postoperative sample was available, ESR at 24 h (ESR(T24)) and the corresponding short-term variation (ΔESR = ESR(T24) − ESR(T0)) were evaluated to characterise early postoperative ESR dynamics. Differences in ESR(T24) and ΔESR between surgical survivors and non-survivors were assessed using non-parametric methods. For the main prognostic comparison between surgical survivors and non-survivors, effect size was estimated using the rank-biserial correlation. All secondary analyses were considered exploratory in nature, and no adjustment for multiple testing was applied.

To investigate potential associations between ESR and the anatomical site of disease, ESR(T0) values were compared between small-intestinal and large-intestinal cases, and the same comparison was performed for ESR(T24) when postoperative values were available. As an exploratory assessment, ESR(T0) values were additionally compared across three clinically meaningful outcome categories, **medically managed survived** horses, surgical survivors and surgical non-survivors, using the Kruskal–Wallis test.

Statistical significance was set at *p* < 0.05 for all analyses. All statistical analyses were performed using IBM SPSS Statistics for Windows, Version 29.0 (IBM Corp., Armonk, NY, USA).

### 2.5. Ethical Approval

Ethical approval for this study was obtained from the Institutional Ethics Committee (Protocol No. 399536/2024).

## 3. Results

### 3.1. Study Population Overview

A total of 85 horses met the inclusion criteria and were included in the analysis. The medically managed survivor group comprised 41 horses with colic that were managed medically and survived to discharge, whereas the surgical group included 44 horses that underwent exploratory celiotomy. Within the surgical group, 27 horses survived to discharge, and 17 were classified as non-survivors due to euthanasia before anaesthesia, intraoperative euthanasia in the presence of non-viable intestine or death/euthanasia within 24 h after surgery.

The demographic and basic clinical characteristics of the population are summarised in Table 1. Age ranged from 2 to 30 years in the **medically managed survivor group** (median 13.0 years, interquartile range [IQR] 7.25–17) and from foals younger than 12 months (reported as 0 years) to 31 years in the surgical group (median 15.5 years, IQR 11–21). In the **medically managed survivor group**, there were 5 entire males, 18 geldings and 18 females; in the surgical group, there were 7 entire males, 25 geldings and 12 females. Breed representation was heterogeneous in both cohorts. In the **medically managed survivor group**, the most frequently recorded categories included Italian Saddle Horses (*n* = 8), Thoroughbred-type horses (*n* = 7), and Quarter Horses or western-type breeds (*n* = 5). Other breeds represented in smaller numbers included Haflingers, Arabians, Pony types, and various crossbreeds. The surgical group also showed a diverse distribution, with Thoroughbred-type horses being the most represented (*n* = 11), followed by Warmblood/Sport Horse types (*n* = 6), and Mixed-breed or crossbred horses (*n* = 4). A variety of additional individual breeds were present in lower frequencies. Regarding the intestinal segment lesions distribution, large-intestinal involvement was diagnosed in 40/41 **medically managed surviving** horses (97.6%) and small-intestinal involvement in 1/41 (2.4%). In the surgical group, 26/44 horses (59.1%) had small-intestinal lesions and 18/44 (40.9%) had large-intestinal lesions. ESR(T0) was available for all 85 horses. Postoperative ESR at 24 h (ESR(T24)) was available for 34 surgically treated horses that remained hospitalised long enough to undergo routine postoperative blood sampling.

### 3.2. ESR at Admission (ESR(T0)) in Medically Managed Survivor Group and Surgical Groups

Admission ESR values (ESR(T0)) varied widely within both groups. Horses in the **medically managed survivor group** (*n* = 41) had a median ESR(T0) of 42 mm/h (IQR 29–59), whereas horses in the surgical group (*n* = 44) showed a lower median of 32.0 mm/h (IQR 13.8–54.5). Extreme ESR values were reviewed and were considered to reflect true biological variability rather than analytical artefacts, as all measurements were performed using a validated point-of-care analyser, within established sample stability limits, and in accordance with internal quality control procedures. Although a trend toward lower ESR(T0) was observed in horses requiring surgery, the difference did not reach statistical significance (Mann–Whitney U, *p* = 0.07; Tables S2–S4).

### 3.3. ESR at Admission (ESR(T0)) in Surgical Survivors and Non-Survivors

**Figure 1:**
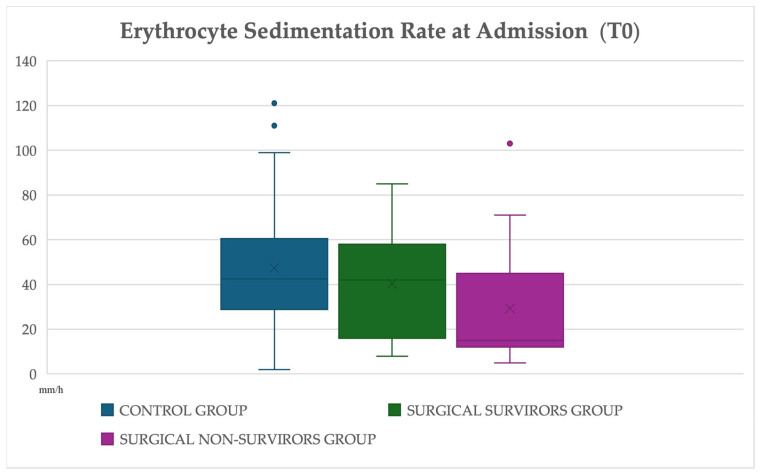
**Distribution of ESR values at admission (ESR(T0)) in medically managed survivors (*n* = 41), surgical survivors (*n* = 27) and surgical non-survivors (*n* = 17).** In each boxplot, the horizontal line represents the median, the box indicates the interquartile range (IQR), and the whiskers represent the full range of observed values. The “X” symbol denotes the mean value. Group comparisons were performed using non-parametric statistical methods; differences across the three outcome categories were assessed using the Kruskal–Wallis test, as described in the Section 2.4 Statistical Analysis.

Within the surgical group, ESR(T0) differed significantly between survivors and non-survivors (Figure 1). Horses that survived to discharge had a median ESR(T0) of 42.0 mm/h (IQR 16.5–56.0), whereas non-survivors presented substantially lower values (15.0 mm/h, IQR 12.0–34.0). This difference was statistically significant (Mann–Whitney U = 128.0, *p* = 0.049), indicating a meaningful association between lower ESR at presentation and poorer outcome in horses requiring surgery (Tables S2 and S4). The magnitude of this difference was moderate, as indicated by the rank-biserial correlation.

### 3.4. ESR(T0) Across Clinical Outcome Categories

When ESR(T0) was compared across **medically managed survivors** (*n* = 41), surgical survivors (*n* = 27) and surgical non-survivors (*n* = 17), significant differences were detected (Kruskal–Wallis H = 7.17, *p* = 0.028) (Supplementary Tables S2–S4) (Figure 1).

### 3.5. ESR at 24 h (ESR(T24)) and Short-Term ESR Evolution

Among the 34 horses for which postoperative ESR measurements were available, ESR(T24) values showed a tendency to increase relative to admission values, particularly in the subgroup of surgical survivors. Among the 17 surgical non-survivors, ESR(T24) was available for 7 horses, whereas the remaining 10 horses were euthanised before anaesthesia, euthanised intraoperatively, or died or were euthanised within 24 h after surgery and therefore did not contribute postoperative ESR measurements. Survivors had a median ESR(T24) of 61.0 mm/h (IQR 44.0–92.0) and a median ΔESR of +26 mm/h (IQR 10.5–44.0). In contrast, the smaller number of non-survivors with postoperative sampling (*n* = 7) had a median ESR(T24) of 54.0 mm/h (IQR 21.5–75.0) and a modest change between admission and 24 h (median ΔESR +1 mm/h, IQR −22.5–40.0).

**Figure 2:**
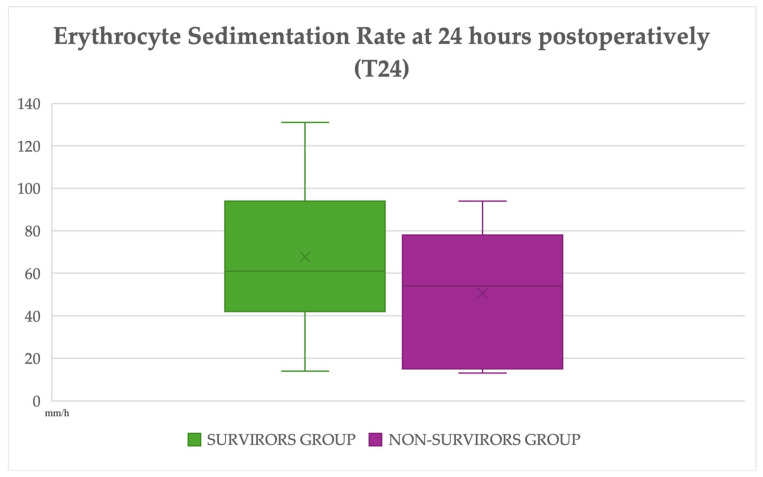
**ESR values measured 24 h postoperatively (ESR (T24)) in surgical survivors (*n* = 27) and surgical non-survivors with available postoperative sampling (*n* = 7).** In each boxplot, the horizontal line represents the median, the box indicates the interquartile range (IQR), and the whiskers represent the full range of observed values. The “X” symbol denotes the mean value. Comparisons between groups were performed using non-parametric statistical methods, as detailed in the Section 2.4 Statistical Analysis.

Neither ESR(T24) nor ΔESR differed significantly between the two outcome groups (*p* = 0.27 and *p* = 0.20, respectively) (Supplementary Tables S2 and S4) (Figure 2).

### 3.6. ESR and Intestinal Segment Involvement

Comparisons of ESR values between the intestinal segments involved did not reveal significant differences. At admission, horses with small-intestinal lesions (*n* = 27) had a median ESR(T0) of 31.0 mm/h (IQR 13–56), while those with large-intestinal lesions (*n* = 58) had a median of 41.0 mm/h (IQR 18–58), with no statistically significant difference (*p* = 0.24, Mann–Whitney U). Among surgical cases with postoperative values, ESR(T24) showed similar distributions between small-intestinal (median 56.5 mm/h) and large-intestinal lesions (median 61.0 mm/h), with no significant difference (*p* = 0.61) (Supplementary Tables S2 and S4).

### 3.7. Exploratory Analyses: Age and Sex

Exploratory evaluation of selected signalment variables did not identify meaningful associations with ESR(T0). No correlation was observed between ESR(T0) and age (Spearman ρ = −0.03, *p* = 0.77), and ESR(T0) values did not differ significantly between male and female horses (*p* = 0.37) (Supplementary Table S2).

## 4. Discussion

This study provides new insight into the behaviour of ESR in horses presenting with colic and its relationship with survival outcomes. The recent validation of a rapid point-of-care ESR analyser for equine use has enabled, for the first time, evaluation of this biomarker in the acute clinical setting. ESR should be regarded as a non-specific, adjunctive parameter that may support early risk stratification when interpreted alongside clinical assessment and established biomarkers, rather than as a stand-alone predictor of outcome.

Using the same analytical method allowed direct comparison with published physiological reference intervals for healthy horses.

Reference intervals were defined as 18.6–100.1 mm/h with a mean of 59.3 mm/h in geldings, 13.8–55.7 mm/h with a mean of 34.8 mm/h in stallions, and 13.9–87.3 mm/h with a mean of 34.0 mm/h in mares [25]. In most inflammatory conditions, ESR would be expected to increase because rising fibrinogen and other acute-phase proteins enhance erythrocyte aggregation. This provides an important physiological backdrop for interpreting ESR values at admission in horses with colic [24,26].

When compared with the physiologic intervals, the ESR(T0) values obtained in our study showed a clear alignment for both medically managed horses and surgical survivors. In the medically managed survivor group, ESR(T0) had a mean of 46.8 mm/h and a median of 42 mm/h (IQR 29–59), while surgical survivors presented a mean of 40.4 mm/h and an identical median of 42 mm/h (IQR 16.5–56). These values fall comfortably within the central portion of the MINI-PET reference distributions. Thus, at the time of admission, ESR values in surviving horses did not deviate markedly from expected physiological values, despite the presence of clinically relevant abdominal disease. This finding is consistent with the known behaviour of ESR in the early stages of inflammation. ESR does not necessarily rise immediately at the onset of the disease, and values may remain within physiological limits unless a more profound systemic inflammatory or haemodynamic disturbance is present [21].

Additionally, medically managed horses predominantly presented with large-intestinal lesions, whereas surgically treated horses included a higher proportion of small-intestinal disease, representing a potential confounding factor. Nevertheless, no significant differences in ESR values were detected between small- and large-intestinal lesions within the study population.

A distinct contrast was observed in surgical non-survivors, whose ESR(T0) values were substantially lower than those of survivors, with a mean of 28.7 mm/h and a median of 15 mm/h (IQR 12–34). This median lies at or below the lower physiological thresholds reported for healthy horses (13.8–18.6 mm/h, depending on sex), highlighting a marked downward shift irrespective of the intestinal segment involved.

It is reasonable to hypothesise that the unexpectedly low ESR in non-survivors may relate to the profound systemic derangements associated with endotoxemia and systemic inflammatory response syndrome (SIRS). These are well-recognised sequelae of severe, life-threatening small- and large-intestinal colic disease [27]. Multiple mechanisms described in the literature may support this interpretation. Endotoxemia and SIRS profoundly affect erythrocyte behaviour, leading to alterations in membrane charge, deformability, and microvascular transit that impair rouleaux formation, an essential determinant of sedimentation velocity [28]. These effects are complemented by alterations in plasma composition. In horses, ESR correlates positively with fibrinogen, plasma viscosity and total serum globulins, and inversely with haematocrit [29]. Early hypovolaemia and fluid sequestration can lead to transient haemoconcentration and increased whole-blood viscosity, both of which slow erythrocyte falls and are recognised negative determinants of ESR in horses and other species [30,31,32].

Furthermore, the acute redistribution or functional impairment of high-molecular-weight plasma proteins during early SIRS, particularly fibrinogen and specific globulin fractions, may limit their ability to neutralise erythrocyte surface charge and promote aggregation, thereby reducing sedimentation despite the presence of substantial systemic inflammation [33].

Taken together, these hemorheological changes provide a physiologically coherent explanation for why ESR may remain unexpectedly low in horses experiencing rapidly progressive, life-threatening colic: the observed depression in ESR likely reflects the severity of systemic disturbance rather than the absence of an inflammatory response. However, because no published studies have directly examined ESR behaviour under these conditions in horses, this interpretation remains inferential and based on established pathophysiological principles rather than disease-specific evidence, highlighting the need for further investigation. Furthermore, these interpretations should be regarded as hypothesis-generating, as no concurrent markers of systemic inflammation, perfusion status, or acute-phase response were measured in the present study.

Understanding these mechanisms also provides a framework for interpreting how ESR changed over the first 24 h following surgery. Surgical survivors showed a clear rise in ESR(T24), with a median of 61 mm/h, a pattern consistent with the delayed activation of the acute-phase response and the expected progression of postoperative inflammation [22,24]. In contrast, postoperative non-survivors demonstrated little or no increase in ESR, a finding that likely reflects the same profound systemic compromise identified at admission. In these horses, as presented above, persistent endotoxemia or progression to severe SIRS may have led to lower values. Accordingly, these proposed mechanisms remain speculative and are intended to provide a physiological framework for interpreting the observed patterns rather than definitive causal explanations.

Some limitations of this study should be acknowledged. The number of non-survivors with postoperative ESR measurements was small, which reduced the power to detect differences in ESR kinetics after surgery. The limited number of non-survivors precluded robust threshold-based analyses, such as receiver operating characteristic curve evaluation. In addition, the interval between the onset of colic signs and hospital admission could not be standardised, meaning that horses were sampled at different points along their inflammatory and haemodynamic trajectory. The heterogeneity of a real-world referral population, including broad variation in lesion types, severities and systemic responses, may also have contributed to variability in ESR values. Another limitation is that ESR(T24) was not sampled for the medically managed survivor group, preventing comparison of short-term ESR evolution between treatment pathways. Furthermore, this study did not perform parallel evaluation of acute-phase proteins or other inflammatory markers, and the exploratory design focused exclusively on ESR without assessing its relationship to established biomarkers of systemic inflammation. Without concurrent assessment of acute-phase proteins, coagulation-related parameters, erythrocyte morphology, or hydration status, it is not possible to determine whether low ESR values primarily reflect SIRS-associated hemorheological alterations, dehydration-related increases in blood viscosity, or a combination of factors. The absence of parallel measurements of established inflammatory and perfusion markers limits mechanistic interpretation and warrants cautious hypothesis-based discussion.

Future work should include larger populations and controlled sampling intervals to validate the prognostic relevance of ESR at admission. Correlating ESR with recognised early prognostic indicators, such as blood lactate, whose marked elevation is strongly associated with poor outcome and mortality in equine colic [16], may help determine whether the markedly low ESR values observed in non-survivors represent an analogous prognostic signal, functioning in a manner comparable to high lactate but reflecting a distinct hemorheological pathway. Parallel measurement of acute-phase proteins, including serum amyloid A and fibrinogen, may also clarify whether ESR reflects inflammatory kinetics distinct from those captured by traditional markers. Finally, serial assessment of ESR over the first 48–72 h, combined with evaluation of haemodynamic stability and SIRS status, could determine whether ESR contributes meaningfully to multimodal prognostic frameworks for early decision-making in horses with colic. Accordingly, the present study should be regarded as an exploratory investigation aimed at describing ESR behaviour in equine colic, rather than at establishing causal mechanisms or definitive prognostic models.

## 5. Conclusions

The results of this study show that ESR measured at admission may provide valuable insight into the expected clinical trajectory and survival outcome of horses presenting with colic. Although most horses exhibited ESR values within the physiological range at presentation, non-survivors demonstrated markedly lower values, often at or below the lower limits reported for healthy individuals. This pattern suggests that low ESR at admission may be a marker of severe systemic disturbance associated with rapidly progressive endotoxemia or SIRS.

Postoperative ESR evolution further supported this interpretation. Survivors showed the expected increase over the first 24 h, whereas non-survivors displayed little or no progression, consistent with profound haemodynamic compromise. Although ESR cannot characterise lesion type and its mechanistic basis in severe colic remains to be clarified, these findings indicate that ESR may contribute prognostic value during early assessment.

Future studies are needed to validate these observations in larger populations, to investigate how ESR relates to acute-phase proteins and to established early prognostic indicators such as blood lactate, and to determine whether ESR can enhance multimodal prognostic frameworks for equine colic.

## Figures and Tables

**Table 1:** **Demographic and clinical characteristics of the study population**. Summary of age distribution, sex, breed representation, intestinal segment involved, outcome and availability of ESR measurements in the **medically managed survivor group** and surgical group (horses undergoing exploratory celiotomy).

Variable	Medically Managed Survivors (*n* = 41)	Surgical (*n* = 44)
**Age, median (IQR)**	13.0 (7.25–17)	15.5 (11–21)
**Age range**	2–30 years	0–31 years
**Sex: Males**	5	7
**Sex: Geldings**	18	25
**Sex: Females**	18	12
**Small-intestinal lesions**	1 (2.4%)	26 (59.1%)
**Large-intestinal lesions**	40 (97.6%)	18 (40.9%)
**Survivors**	41 (100%)	27 (61.4%)
**Non-survivors**	0 (0%)	17 (38.6%)
**Most frequent breeds**	Warmblood (*n* = 8), Thoroughbred (*n* = 7), Quarter Horse type (*n* = 5)	Thoroughbred (*n* = 11), Warmblood (*n* = 6), Mixed (*n* = 4)
**ESR(T0) available**	41	44
**ESR(T24) available**	–	34

All descriptive statistical analyses are reported in Supplementary Table S1.

## Data Availability

The data supporting the findings of this study are not publicly available due to privacy and institutional restrictions, but may be provided by the corresponding author upon reasonable request.

## Supplementary Materials

The following supporting information can be downloaded at: https://www.mdpi.com/article/10.3390/ani16030476/s1, Table S1: Descriptive statistics for ESR(T0), ESR(T24), and ΔESR across study groups; Table S2: Summary of inferential statistical analyses; Table S3: ESR T0 Values in the medically managed survivor group; Table S4: Raw ESR Data for Surgical Horses.
