# Supplementary material for: Prognostic Significance of Erythrocyte Sedimentation Rate for Survival in Equine Colic
Source: Animals (Basel). 2026 Feb 3;16(3):476. doi: 10.3390/ani16030476 (PMC12896511; doi:10.3390/ani16030476)
Supplement: Supplementary file 1 [file animals-16-00476-s001.zip › animals-4089597-supplementary.pdf]

Supplementary Materials

Group	Variable	n	Mean	SD	Median	IQR 25	IQR 75	Min	Max
**Control**	ESR(T0)	41	46.80	27.92	42.0	29.0	59.0	2.0	121.0
**Control**	ESR(T24)	0	—	—	—	—	—	—	—
**Control**	ΔESR	0	—	—	—	—	—	—	—
**Surgical survivors**	ESR(T0)	27	40.37	22.61	42.0	16.5	56.0	8.0	85.0
**Surgical survivors**	ESR(T24)	27	67.74	31.25	61.0	44.0	92.0	14.0	131.0
**Surgical survivors**	ΔESR	27	27.37	31.06	26.0	10.5	44.0	−34.0	103.0
**Surgical non-survivors**	ESR(T0)	17	28.71	27.29	15.0	12.0	34.0	5.0	103.0
**Surgical non-survivors**	ESR(T24)	7	50.57	32.39	54.0	21.5	75.0	13.0	94.0
**Surgical non-survivors**	ΔESR	7	7.43	45.22	1.0	−22.5	40.0	−49.0	65.0

Supplementary Table S1. Descriptive statistics for ESR(T0), ESR(T24), and ΔESR across study groups.
Mean, standard deviation, median, IQR and range for ESR variables in control horses, surgical survivors and surgical non-survivors.

Comparison	Test	Statistic	p-value	n(1)	n(2)
**ESR(T0) Control vs Surgical**	Mann–Whitney	U = 1108.5	0.0699	41	44
**ESR(T0) Surgical survivors vs non-survivors**	Mann–Whitney	U = 311.5	0.0493	27	17
**ESR(T0) across 3 categories**	Kruskal–Wallis	H = 7.17	0.0278	41	44
**ESR(T24) survivors vs non-survivors**	Mann–Whitney	U = 63.0	0.2679	27	7
**ΔESR survivors vs non-survivors**	Mann–Whitney	U = 62.0	0.2012	27	7

**ESR(T0) small vs large intestine**	Mann–Whitney	U = 652.0	0.2754	27	58
**ESR(T24) small vs large intestine**	Mann–Whitney	U = 111.0	0.6117	20	14
**ESR(T0) vs age**	Spearman	$\rho = -0.0497$	0.6515	85	—
**ESR(T0) vs sex (M/MC vs F)**	Mann–Whitney	U = 398.0	0.4451	55	30

Supplementary Table S2. Summary of inferential statistical analyses.
Non-parametric comparisons (Mann–Whitney U, Kruskal–Wallis) and correlation tests performed on ESR variables, including sample size, test statistic and p-values.

Horse_ID	Group	ESR(T0) (mm/h)
**1**	1	42
**2**	1	38
**3**	1	15
**4**	1	59
**5**	1	63
**6**	1	72
**7**	1	48
**8**	1	44
**9**	1	29
**10**	1	11
**11**	1	121
**12**	1	39
**13**	1	55
**14**	1	18
**15**	1	51
**16**	1	33
**17**	1	59
**18**	1	39
**19**	1	32
**20**	1	23
**21**	1	52
**22**	1	51
**23**	1	47
**24**	1	71
**25**	1	46
**26**	1	68
**27**	1	30
**28**	1	71
**29**	1	49
**30**	1	44
**31**	1	61

**32**	1	29
**33**	1	44
**34**	1	17
**35**	1	12
**36**	1	52
**37**	1	40
**38**	1	17
**39**	1	10
**40**	1	21
**41**	1	25

Supplementary S3: ESR T0 Values in the control group

Horse_ID	ESR(T0)	ESR(T24)	ΔESR	Outcome (1/2)	Segment (1=SI,2=LI)
**1**	14	—	—	2	2
**2**	29	52	+23	1	2
**3**	54	54	0	1	2
**4**	62	116	+54	1	2
**5**	13	116	+103	1	1
**6**	69	90	+21	1	1
**7**	29	61	+32	1	2
**8**	5	—	—	2	2
**9**	56	—	—	2	2
**10**	72	38	–34	1	1
**11**	34	—	—	2	1
**12**	85	131	+46	1	2
**13**	15	—	—	2	2
**14**	13	32	+19	1	2
**15**	12	—	—	2	1
**16**	8	14	+6	1	1
**17**	15	—	—	2	2
**18**	13	—	—	2	2
**19**	29	48	+19	1	2
**20**	34	—	—	2	1
**21**	56	34	–22	1	1
**22**	31	—	—	2	1
**23**	41	53	+12	1	2
**24**	20	—	—	2	1
**25**	23	—	—	2	1
**26**	13	13	0	1	2
**27**	65	—	—	2	1
**28**	19	33	+14	1	2
**29**	12	—	—	2	2
**30**	12	24	+12	1	2
**31**	26	41	+15	1	2
**32**	8	—	—	2	1
**33**	13	26	+13	1	2

**34**	45	90	+45	1	2
**35**	16	—	—	2	1
**36**	20	46	+26	1	2
**37**	46	—	—	2	2
**38**	61	61	0	1	2
**39**	56	94	+38	1	1
**40**	28	—	—	2	1
**41**	13	13	0	1	2
**42**	23	—	—	2	2
**43**	14	—	—	2	2
**44**	16	13	–3	1	1

Supplementary Table S4: Raw ESR Data for Surgical Horses: Raw ESR(T0), ESR(T24) and short-term ESR change (ΔESR) for all surgically treated horses (n = 44), anonymised using Horse_ID corresponding to the sequential case number in the dataset. Outcome: 1 = survivor; 2 = non-survivor. Segment: 1 = small intestine; 2 = large intestine.
